# Reduced Inter-hemispheric Resting State Functional Connectivity and Its Association With Social Deficits in Autism

**DOI:** 10.3389/fpsyt.2021.629870

**Published:** 2021-03-04

**Authors:** Shuxia Yao, Benjamin Becker, Keith M. Kendrick

**Affiliations:** The Clinical Hospital of Chengdu Brain Science Institute, MOE Key Laboratory for NeuroInformation, Center for Information in Medicine, University of Electronic Science and Technology of China, Chengdu, China

**Keywords:** autism spectrum disorder, resting state, inter-hemispheric functional connectivity, homotopic connectivity, corpus callosum, social deficits, biomarker

## Abstract

Autism spectrum disorder (ASD) is an early onset developmental disorder which persists throughout life and is increasing in prevalence over the last few decades. Given its early onset and variable cognitive and emotional functional impairments, it is generally challenging to assess ASD individuals using task-based behavioral and functional MRI paradigms. Consequently, resting state functional MRI (rs-fMRI) has become a key approach for examining ASD-associated neural alterations and revealed functional alterations in large-scale brain networks relative to typically developing (TD) individuals, particularly those involved in social-cognitive and affective processes. Recent progress suggests that alterations in inter-hemispheric resting state functional connectivity (rsFC) between regions in the 2 brain hemispheres, particularly homotopic ones, may be of great importance. Here we have reviewed neuroimaging studies examining inter-hemispheric rsFC abnormities in ASD and its associations with symptom severity. As an index of inter-hemispheric functional connectivity, we have additionally reviewed previous studies on corpus callosum (CC) volumetric and fiber changes in ASD. There are converging findings on reduced inter-hemispheric (including homotopic) rsFC in large-scale brain networks particularly in posterior hubs of the default mode network, reduced volumes in the anterior and posterior CC, and on decreased FA and increased MD or RD across CC subregions. Associations between the strength of inter-hemispheric rsFC and social impairments in ASD together with their classification performance in distinguishing ASD subjects from TD controls across ages suggest that the strength of inter-hemispheric rsFC may be a more promising biomarker for assisting in ASD diagnosis than abnormalities in either brain wide rsFC or brain structure.

## Introduction

Autism spectrum disorder (ASD) is an early onset developmental disorder which persists throughout life and has an increasing prevalence rate over the last decades, with a general average of around 1% of the population being affected (1). Although symptoms of ASD are highly heterogeneous, the disorder is generally characterized by deficits mainly in social communication/interaction, restricted interests, and repetitive behaviors (2). Task-based behavioral and neuroimaging studies have further shown specific behavioral deficits and corresponding neural dysfunctions in ASD primarily in social-cognitive and affective domains, such as facial emotion recognition (3–5), eye contact avoidance (6–8), and social interest (9, 10).

However, given the early onset of the disorder and general cognitive deficits in ASD, assessment of ASD individuals, particularly young children, by means of task-based behavioral and fMRI paradigms is challenging. Consequently, resting state fMRI (rs-fMRI) has been proposed as a promising approach for examining ASD-associated alterations. Rs-fMRI assesses subjects in the absence of a specific task and thus allows comparisons across different studies and data acquisition sites. Previous rs-fMRI studies have reported functional abnormalities in large-scale brain networks in ASD relative to typically developing (TD) individuals (11–14), particularly in brain networks involved in social-cognitive and affective processes (15–17). Importantly, rs-fMRI measurement across different sites and research groups allows the collection of large sample sizes and data sharing initiatives, such as the Autism Brain Imaging Data Exchange (ABIDE) databases (ABIDE-I and -II), although it should be acknowledged that these span wide age ranges and are restricted to individuals with high-functioning ASD. As a result of increased statistical power using these large databases, previous studies have demonstrated more consistent and reproducible patterns of alterations in resting state functional connectivity (rsFC) in ASD populations (11, 18, 19).

It has been shown that functional connectivity alterations in psychiatric disorders are mainly contributed by counterpart regions in the opposite hemisphere (20). This is also the case in ASD where rsFC alterations are primarily contributed by inter-hemispheric connectivity between regions in the 2 hemispheres (11, 21), and homotopic ones (between identical regions in the 2 hemispheres) may be of particular importance (19, 22, 23). The aims of the present review are to detail findings from neuroimaging studies directly examining interhemispheric rsFC abnormalities in ASD (see Table 1), particularly the homotopic ones together with their associations with symptom severity, and studies investigating structural changes in the corpus callosum (CC) in ASD (see Table 2 for volumetric studies and Table 3 for diffusion-weighted imaging (DWI) studies), which is also an important index of interhemispheric functional connectivity. We also discussed their functional significance and whether they can be used as promising biomarkers for future clinical application.

**Table 1:** Overview of the studies examining alterations in interhemispheric (homotopic) functional connectivity in ASD patients.

**References**	**Autism**					**Control**					**Method**	**Conclusion**
	***N* (F)**	**Age**	**VIQ**	**PIQ**	**FIQ**	***N* (F)**	**Age**	**VIQ**	**PIQ**	**FIQ**		
Anderson et al. (22)	53 (0)	22.40 (7.20)	101.30 (21.10)	101.30 (16.50)	/	39 (0)	21.10 (6.50)	116.00 (14.80)	114.20 (13.90)	/	Voxel-Based whole brain	↓^*^
Cheng et al. (11)	418 (51)	17.17 (7.97)	104.50 (15.87)	105.30 (15.17)	105.7 (14.16)	509 (85)	16.40 (7.08)	111.10 (12.84)	107.24 (12.56)	110.62 (11.93)	Voxel-Based whole brain	↓
Dickinson et al. (24)	59 (13)	5.79 (2.01)	68.96 (34.35)	74.67 (33.82)	/	39 (13)	5.96 (2.22)	121.12 (19.42)	112.55 (12.07)	/	EEG coherence analysis	↓
Dickinson et al. (25)	35 (9)	12/24 months	/	/	/	20 (11)	12/24 months	/	/	/	EEG coherence analysis	↓
Di Martino et al. (19)	360 (0)	16.30 (7.00)	105.00 (16.00)	106.00 (15.00)	105.00 (16.00)	403 (0)	16.30 (7.00)	111.00 (13.00)	108.00 (12.00)	111.00 (11.00)	Voxel-Based whole brain	↓^*^
Dinstein et al. (26)	29 (/)	29 months (/)	/	/	/	30 (/)	28 months (/)	/	/	/	ROI analysis	↓^*^
Guo et al. (27)	105 (0)	10.15 (1.26)	/	/	110.53 (17.42)	102 (0)	10.02 (1.38)	/	/	113.78 (11.98)	Sliding-Window FC analysis	↓
Hahamy et al. (28)	68 (6)	26.6 (1.87)	105.79 (7.28)	108.42 (6.35)	107.76 (7.56)	73 (14)	25.82 (2.02)	113.27 (2.95)	111.67 (2.62)	114.14 (2.48)	Voxel-Based whole brain	↓ and ↑^*^
Keehn et al. (29)	27 (/)	3/6/9/12 months	/	/	/	37 (/)	3/6/9/12 months	/	/	/	fNIRS ROI analysis	↓
King et al. (30)	579 (373)	15.1 (6.9)	105.70 (17.93)	105.18 (17.06)	106.05 (16.79)	823 (463)	15.1 (6.8)	113.85 (13.48)	109.43 (13.64)	113.03 (12.53)	ROI analysis	↓^*^
Lazarev et al. (31)	14 (0)	9.70 (2.20)	91.00 (27.50)	94.30 (20.40)	91.40 (22.80)	19 (0)	10.10 (3.46)	/	/	/	EEG coherence analysis	↓
Lee et al. (21)	458 (54)	16.2 (7.4)	/	/	106.0 (16.3)	517 (90)	16.50 (7.30)		/	111.20 (12.40)	Voxel-Based whole brain	↓
Li et al. (23)	409 (47)	17.42 (8.56)	105.20 (18.10)	106.27 (16.81)	106.72 (16.65)	455 (72)	17.35 (7.76)	111.48 (13.53)	108.17 (13.46)	111.45 (12.55)	Voxel-Based whole brain	↓^*^
Yao et al. (32)	146 (25)	8.48 (1.07)	107.80 (19.37)	107.27 (20.15)	106.03 (19.40)	175 (47)	8.62 (0.84)	115.09 (15.89)	109.35 (15.40)	114.43 (13.40)	Voxel-Based whole brain	↓^*^
Zhu et al. (33)	10 (0)	9.00 (1.30)	/	/	/	10 (0)	8.90 (1.40)	/	/	/	fNIRS ROI analysis	↓

For age and IQs, mean and standard deviation are reported. N (F): Number of subjects and female subjects; VIQ, Verbal IQ; PIQ, Performance IQ; FIQ, Full IQ; EEG, electroencephalographic; ICA, Independent component analysis; ↑ or ↓, Increased or decreased rsFC in autism subjects compared to typically developing subjects;

* *Homotopic functional connections*.

**Table 2:** Overview of the studies examining alterations in corpus callosum volume in ASD patients.

**References**	**Autism**					**Control**					**Method**	**Conclusion**
	***N* (F)**	**Age**	**VIQ**	**PIQ**	**FIQ**	***N* (F)**	**Age**	**VIQ**	**PIQ**	**FIQ**		
Anderson et al. (22)	53 (0)	22.40 (7.20)	101.30 (21.10)	101.30 (16.50)	/	39 (0)	21.10 (6.50)	116.00 (14.80)	114.20 (13.90)	/	SBM	↓
Chung et al. (34)	16 (0)	16.10 (4.50)	/	/	/	12 (0)	17.10 (2.80)	/	/	/	VBM	↓
Egaas et al. (35)	51 (6)	15.50 (10.0)	<70:16; ≥70:31	/	/	51 (6)	15.50 (9.90)	/	/	/	TMA	↓
Elia et al. (36)	22 (0)	10.92 (4.02)	/	/	/	11 (0)	10.86 (2.85)	/	/	/	TMA	N.S.
Frizer et al. (37)	253 (27)	14.58 (6.00)	/	/	/	250 (53)	14.41 (5.31)	/	/	/	Meta	↓
Gaffney et al. (38)	13 (3)	11.30 (4.70)	/	/	84.9 (26.7)	35 (14)	12.00 (5.20)	/	/	/	TMA	N.S.
Haar et al. (39)	295 (0)	16.89 (8.10)	/	/	106.16 (17.16)	295 (0)	16.72 (7.55)	/	/	111.21 (12.15)	SBM	↓
Hardan et al. (40)	22 (0)	22.40 (10.10)	103.00 (16.40)	97.50 (12.60)	100.40 (14.70)	22 (0)	22.40 (10.00)	100.50 (14.70)	99.60 (12.50)	100.50 (14.20)	TMA	↓
Lefebvre et al. (41)	328 (38)	16.60 (8–39)	103.00 (18.00)	106.00 (17.00)	105.00 (17.00)	366 (62)	17.00 (8–40)	111.00 (13.00)	107.00 (13.00)	111.00 (12.00)	SBM	N.S.
Li et al. (23)	409 (47)	17.42 (8.56)	105.20 (18.10)	106.27 (16.81)	106.72 (16.65)	455 (72)	17.35 (7.76)	111.48 (13.53)	108.17 (13.46)	111.45 (12.55)	VBM	↓
Manes et al. (42)	27 (5)	14.35 (6.80)	/	/	/	17 (6)	11.85 (5.00)	/	/	/	TMA	↓
Piven et al. (43)	35 (9)	18.00 (4.50)	/	91.00 (19.80)	/	36 (16)	20.20 (3.80)	/	102.10 (12.80)	/	TMA	↓
Rice et al. (44)	12 (0)	12.42 (4.32)	106.00 (33.01)	107.83 (17.66)	107.33 (26.06)	8 (0)	12.50 (3.46)	123.25 (10.90)	120.38 (10.08)	124.00 (11.28)	TMA	N.S.
Sui et al. (45)	16 (0)	21.38 (2.39)	/	/	108.88 (17.39)	17 (0)	21.71 (2.14)	/	/	116.65 (11.98)	DKI	↓
Vidal et al. (46)	24 (0)	10.00 (3.30)	92.90 (13.30)	99.10 (14.00)	95.90 (11.50)	26 (0)	11.00 (2.50)	105.40 (11.20)	104.10 (14.00)	104.80 (11.70)	TMA	↓
Waiter et al. (47)	15 (0)	15.20 (2.20)	100.60 (24.20)	99.40 (19.50)	100.50 (22.40)	16 (0)	15.50 (1.60)	101.30 (21.10)	98.80 (13.90)	99.70 (18.30)	VBM	↓
Wolff et al. (48)	57 (10)	6.60 (0.7)	/	/	79.80 (17.60)	108 (42)	6.70 (0.7)	/	/	110.90 (16.00)	Fourier contour model	↑
		12.90 (0.8)	/	/			12.70 (0.7)	/	/			↑
		24.80 (1.2)	/	/			24.70 (0.8)	/	/			N.S.
Yao et al. (32)	146 (25)	8.48 (1.07)	107.80 (19.37)	107.27 (20.15)	106.03 (19.40)	175 (47)	8.62 (0.84)	115.09 (15.89)	109.35 (15.40)	114.43 (13.40)	VBM	N.S.

*For age and IQs, mean and standard deviation are reported. N (F): Number of subjects and female subjects; VIQ, Verbal IQ; PIQ, Performance IQ; FIQ, Full IQ; SBM, Surface-based morphometric analysis; TMA, Traditional morphometric analysis; VBM, Voxel-based morphometric analysis; DKI, diffusional kurtosis imaging; ↑ or ↓, Increased or decreased corpus callosum volume in autism subjects compared to typically developing subjects; N.S, No significant group difference*.

**Table 3:** Overview of the diffusion-weighted imaging studies examining corpus callosum abnormities in ASD patients.

**References**	**Autism**					**Control**					**Method**	**Conclusion**			
	***N* (F)**	**Age**	**VIQ**	**PIQ**	**FIQ**	***N* (F)**	**Age**	**VIQ**	**PIQ**	**FIQ**		**FA**	**MD**	**RD**	**AD**
Alexander et al. (49)	43 (0)	16.23 (6.70)	/	107.49 (13.04)	/	34 (0)	16.44 (5.97)	/	112.79 (12.08)	/	ROI	↓	↑	↑	N.S.
Ameis et al. (50)	71 (15)	11.40 (3.40)	/	/	95.0 (19.7)	62 (25)	10.80 (2.80)	/	/	112.5 (17.1)	TBSS	↓	N.S.	N.S.	N.S.
Aoki et al. (51)	567 (81)	14.23 (/)	/	/	106.42 (/)	482 (116)	15.47 (/)	/	/	111.94 (/)	Meta	↓	↑	/	/
Aoki et al. (52)	69 (7)	8.90 (1.70)	108 (16)	110 (19)	109 (17)	50 (12)	9.40 (1.50)	114 (15)	114 (14)	114 (13)	TBSS	↓	↑	↑	↑
Barnea-Goraly et al. (53)	7 (0)	14.60 (3.40)	84 (17)	121.5 (8)	101 (12.2)	9 (0)	13.40 (2.80)	105.2 (11.4)	107 (10)	107 (8.5)	Voxel based whole brain	↓	/	/	/
Barnea-Goraly et al. (54)	13 (2)	10.50 (2.00)	/	/	85.9 (17.4)	11 (2)	9.60 (2.10)	/	/	119.9 (13.3)	TBSS	↓	/	N.S.	↓
Bashat et al. (55)	17 (/)	1.8–3.3	/	/	/	41 (18)	0.25–23	/	/	/	ROI	↑	/	/	/
Brito et al. (56)	8 (0)	9.53 (1.83)	/	/	/	8 (0)	9.57 (1.36)	/	/	/	ROI	↓	/	/	/
Di et al. (57)	297 (28)	22.40 (/)	/	/	107.87 (/)	302 (30)	22.2 (/)	/	/	112.50 (/)	Meta	↓	/	/	/
Groen et al. (58)	17 (3)	14.40 (1.60)	97 (19)	100 (15)	98 (18)	25 (3)	15.50 (1.80)	105 (10)	105 (11)	105 (9)	Voxel based whole brain	↓	↑	/	/
Hong et al. (59)	18 (0)	8.69 (2.18)	/	/	105.22 (21.12)	16 (0)	9.81 (1.91)	/	/	106.13 (20.13)	ROI	N.S.	/	/	/
Keller et al. (60)	34 (0)	18.90 (7.30)	/	/	102.0 (14.8)	31 (0)	18.90 (6.20)	/	/	109.5 (9.0)	Voxel based whole brain	↓	/	/	/
Kumar et al. (61)	32 (3)	5.00 (/)	/	/	/	16 (4)	5.50 (/)	/	/	/	TBSS	↓	/	/	/
Nickel et al. (62)	30 (11)	35.40 (9.07)	/	/	124.5 (12.26)	30 (11)	35.53 (8.30)	/	/	123.63 (13.80)	Voxel based whole brain	↓	N.S.	/	/
Noriuchi et al. (63)	7 (1)	13.96 (2.68)	93.7 (7.41)	93.14 (8.07)	92.71 (6.68)	7 (1)	13.36 (2.74)	/	/	116.43 (9.50)	Voxel based whole brain	↓	/	/	/
Shukla et al. (64)	26 (2)	12.70 (0.60)	105.6 (3.6)	109.5 (3.3)	/	24 (1)	13.00 (0.60)	108.2 (2.6)	110.3 (2.5)	/	ROI	↓	↑	↑	N.S
Shukla et al. (65)	26 (1)	12.80 (0.60)	104.3 (3.4)	108.8 (3.3)	/	24 (1)	13.00 (0.60)	108.2 (2.6)	110.3 (2.5)	/	TBSS	↓	↑	↑	N.S.
Thomas et al. (66)	12 (0)	28.50 (9.70)	105.08 (10.09)	107.25 (13.9)	106.92 (10.47)	18(0)	22.40 (4.10)	109.8 (12.54)	111 (10.03)	111.6 (9.91)	ROI	N.S.	/	/	/
Travers et al. (67)	100 (0)	18.30 (8.50)	96.3 (21.1)	102.6 (17.9)	100.1 (17.6)	56 (0)	18.90 (7.80)	114.7 (13.2)	116.3 (14.8)	118.2 (13.2)	ROI	↓	↑	↑	↑
Vogan et al. (68)	61 (10)	10.90 (2.00)	/	/	102.2 (14.9)	69 (18)	11.10 (2.40)	/	/	112.7 (11.6)	TBSS	↓	N.S.	N.S.	↓
Walker et al. (69)	39 (11)	4.63 (1.76)	/	/	/	39 (13)	4.74 (1.76)	/	/	/	TBSS	↓	↑	↑	↑
Weinstein et al. (70)	22 (/)	3.20 (1.10)	/	/	/	32 (/)	3.40 (1.30)	/	/	/	TBSS	↑	N.S.	↓	N.S.

*For age and IQs, mean and standard deviation are reported. N (F): Number of subjects and female subjects; VIQ, Verbal IQ; PIQ, Performance IQ; FIQ, Full IQ; TBSS, Tract based spatial statistics; ROI, Region of interest; FA, Fractional anisotropy; MD, Mean diffusivity; RD, Radial diffusivity; AD, Axial diffusivity; ↑ or ↓, Increased or decreased in autism subjects compared to typically developing subjects; N.S, No significant group difference*.

## Inter-hemispheric rsFC Abnormalities in ASD

Based on the cortical underconnectivity theory, aberrant rsFC patterns of ASD can be characterized by local over-connectivity but long-distance under-connectivity (13, 71, 72). As a typical long-distance connection, inter-hemispheric rsFC reflects the correlation of fMRI BOLD signal time series between brain regions belonging to the 2 brain hemispheres and can be used as an important index for delineating inter-hemispheric synchronization abnormalities in spontaneous neural activity in ASD. Support for the functional importance of inter-hemispheric connections comes from studies on agenesis or sections of CC. The CC is the major white-matter tract between the left and right hemispheres and plays a crucial role in the maintenance of interhemispheric functional communications (73). Individuals with congenital callosal agenesis have been reported to exhibit ASD-like symptoms such as deficits in language comprehension, theory of mind and social reasoning (74) and strongly elevated autistic traits as measured by the Autism Spectrum Quotient (75). Reduced inter-hemispheric rsFC in brain regions of the default mode network (DMN) and salience network have also been found in callosal dysgenesis subjects (76), although interestingly this was not found to influence the qualitative organization pattern of the rsFC (76). However, another study has reported an intact pattern of inter-hemispheric rsFC in callosal dysgenesis individuals using region of interest (ROI) and independent component analysis-based approaches (77). This inconsistency could be due to the occurrence of a remarkable plasticity compensating for the disconnection, as revealed by an animal callosotomy study reporting that a restoration of inter-hemispheric rsFC occurred from 7 to 28 days post-callosotomy but only when the callosotomy was partial and not when it was total (78). The human callosal dysgenesis studies reporting normal interhemispheric rsFC would similarly have been partial. Furthermore, another gene knockdown study in rodents has further emphasized the importance of inter-hemispheric connectivity in ASD symptomatology, such that permanent deficits in callosal projections and associated inter-hemispheric connections caused by downregulation of Wnt signaling induced ASD-like impairments, including social interaction deficits and repetitive motor behavior (79). Overall these studies both emphasize the importance of inter-hemispheric connections in the context of normal social functioning and autism and that under normal circumstances these functional connections can be restored to a large extent even when the CC does not develop fully or is partially disconnected.

Based on a voxel-based whole brain analysis, Cheng et al. (11) revealed that the majority of altered rsFC occur between regions from the opposite hemispheres using the ABIDE database, and more specifically that reduced rsFC is found mainly between brain regions implicated in face expression processing, theory of mind and self-other distinction. Also using the ABIDE database, another voxel-based study using a graph theory analysis approach specifically examined inter- and intra-hemispheric rsFC in ASD subjects and reported that they had reduced inter and intra-hemispheric functional connectivity strength in regions of the DMN and the visual system relative to TD subjects (21). Note that significant correlations were only observed between symptom severity, as measured by the restricted and repetitive behavior subscale of the Autism Diagnostic Observation Schedule (ADOS), and the density of inter- but not intra-hemispheric FC in the posterior cingulate cortex (PCC) and precuneus which both represent core nodes of the posterior DMN. Using only data from children included in the ABIDE database, a more recent study using a sliding-window analysis has shown increased inter- and intra-hemispheric dynamic FC variability in the medial pre-frontal cortex and the anterior cingulate cortex but decreased inter- and intra-hemispheric dynamic FC variability in the fusiform gyrus and the inferior temporal gyrus (ITG) in ASD relative to TD children (27). Furthermore, again only aberrant temporal variability of the inter- but not intra-hemispheric FC was associated with ADS symptom load, specifically social communication deficits as measured by the ADOS communication subscale. Overall, these findings tend to indicate that, although inter- and intra-hemispheric rsFC both show abnormities in ASD, the inter-hemispheric ones may be of greater clinical relevance.

Given that atypical developmental trajectories of functional and structural organization in these brain networks have also been demonstrated by longitudinal studies (72, 80, 81), attention should also be paid to the neurodevelopmental trajectories of inter-hemispheric functional connectivity, but current studies on this are very limited. Using functional near infrared spectroscopy (fNIRS), a longitudinal study on infants with a high risk of ASD in the first year of life has demonstrated that while they show an increase in both the inter- and intra-hemispheric connectivity in temporal and inferior frontal cortex at the age of 3 months, they exhibit a pattern of decreased functional connectivity at the age of 12 months (29). Another fNIRS study using similar regions of interest reported that this pattern of decreased inter-hemispheric functional connectivity was maintained in older children (mean age = 9.0 ± 1.3) (33), although no longitudinal study to date has specifically investigated the developmental trajectories of interhemispheric rsFC for children between the ages of 1 to 9 years of age. A recent study using electroencephalographic (EEG) measurement on alpha phase coherence has also revealed reduced inter-hemispheric connectivity in the temporal cortex in ASD children (mean age = 5.79 ± 2.01) (24). A similar pattern of reduced inter-hemispheric connection has also been found between temporal and parietal cortex in tuberous sclerosis complex infants who later developed ASD, which becomes to be most robust at 24 months (25), although the comorbidities of tuberous sclerosis complex and ASD have to be kept in mind when interpreting these findings. Finally, another EEG study with intermittent photic stimulation on an older sample size (mean age = 9.7 ± 2.2) revealed a more general decrease in inter-hemispheric coherent connections of the 20 highest connections at all the beta frequencies corresponding to photic stimulation (31).

## Altered Homotopic Inter-hemispheric rsFC in ASD

Homotopic inter-hemispheric rsFC is a component part of overall interhemispheric rsFC and is an important measure reflecting activity synchronization between homotopic regions of the 2 hemispheres (82, 83). Homotopic FC can be measured using voxel-mirrored connectivity analysis on the correlation of fMRI BOLD signal time series between each voxel in 1 hemisphere and its mirrored counterpart in the other side (82). Homotopic inter-hemispheric rsFC abnormalities have been found in a number of psychiatric disorders including schizophrenia (84), major depression (85), substance dependence (86), and even Parkinson's disease (87).

**Figure 1:**
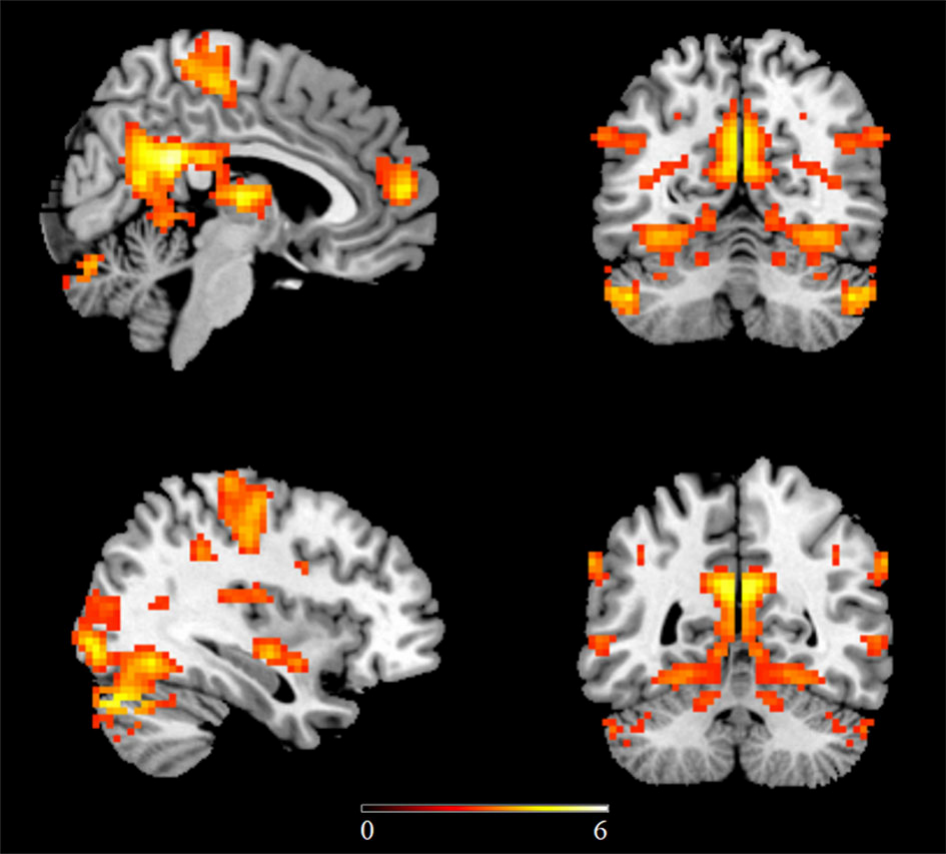
Regions showing decreased homotopic inter-hemispheric functional connectivity in ASD relative to matched typically developing controls using the ABIDE database. Statistic maps were displayed with a *p* < 0.05 FDR corrected threshold. Color bar indicates *t* values of the statistical map. This figure is adapted from Li et al. (23).

In the first study examining homotopic inter-hemispheric rsFC changes in ASD, a moderate sample size (53 high-functioning ASD males vs. 39 TD controls) was used and found decreased homotopic rsFC between the bilateral sensorimotor cortex, anterior insula, and superior parietal lobule in ASD subjects compared to TD controls (22). A further ROI-based study has also revealed decreased homotopic inter-hemispheric rsFC between the superior temporal gyrus (STG) and inferior frontal gyrus (IFG) in naturally sleeping toddlers with autism relative to TD controls (29 ASD vs. 30 controls), with homotopic connectivity strengths of the IFG being negatively correlated with social and communication impairments, as measured by ADOS (26). However, while decreased homotopic connectivity was found in the lateral occipital cortex and the postcentral gyrus in ASD, Hahamy et al. (28) additionally demonstrated increased homotopic inter-hemispheric rsFC in the ITG and the middle frontal gyrus in ASD using a moderate sample size (68 ASD males vs. 73 TD controls) consisting of subjects from selective sites in ABIDE-I. This study has further revealed significantly greater individual topographical distortions in the homotopic rsFC patterns in ASD relative to TD controls (28). More robust findings have subsequently come from studies using larger numbers of subjects from the ABIDE database. In the first study using the ABIDE database, reduced homotopic inter-hemispheric rsFC has also been found in regions including the PCC, insula, and thalamus (19). In a further study which included gender as an additional covariate, Li et al. (23) carried out a voxel-based whole brain analysis on differences in homotopic inter-hemispheric rsFC in ASD subjects and further examined associations between homotopic rsFC and symptom severity. Results showed decreased homotopic rsFC of regions in large-scale brain networks including the DMN, salience network, mirror neuron/motor systems, and auditory and visual systems in ASD adolescents and adults (Figure 1), with homotopic rsFC in the PCC, insula and STG showing significant associations with social impairments as measured by ADOS. A more recent follow-up study using independent data from children in ABIDE I and ABIDE II databases and the same analysis approach has revealed consistent patterns of reduced homotopic inter-hemispheric rsFC in ASD children between the ages of 5 and 10 years old, suggesting that the homotopic alterations can occur at an early age and remain stable in contrast to other dysregulations which vary across developmental periods. Moreover, this study has also demonstrated significant associations between ASD symptom severity and homotopic inter-hemispheric rsFC changes in regions of the DMN (the PCC and the precuneus) and the visual processing networks (inferior and middle temporal gyri and inferior occipital gyrus) (32). Atypical developmental trajectories of homotopic rsFC and its association with symptom severity have also been reported in ASD (88). Note that although a recent study specifically examining reproducibility across sites has also demonstrated overall decreased homotopic inter-hemispheric rsFC in ASD using the whole ABIDE datasets, as consistently observed in most of the previous studies, the decreased homotopic rsFC pattern was not generally reproducible across sites (30), which could be due to decreased statistical power as a result of smaller sample sizes when individual sites were examined separately.

## Interhemispheric rsFC—a More Promising Biomarker for ASD?

In both seed-based and voxel-based rsFC analyses, altered rsFC in the DMN, particularly the PCC, is one of the most replicated findings in ASD samples (12, 13, 19, 89–92). As we outlined above, this is also the case for interhemispheric rsFC, particularly the homotopic connections (19, 21, 23, 32). Interestingly, the PCC and precuneus of the DMN, the STG, and the insula which exhibited associations between their interhemispheric rsFC (including the homotopic ones) and symptom severity in ASD, are all regions strongly involved in social and affective processing (21, 23, 32). These associations have been demonstrated only for the inter-hemispheric (including the homotopic) rsFC alterations but not for the intra-hemispheric ones (21, 23, 27). Furthermore, based on inter-hemispheric, but not only the homotopic rsFC changes, pattern classification analysis achieved an average accuracy of 88.70% for distinguishing ASD subjects from TD controls across sites (23), which is slightly higher than a previous study (82.87%) using the same pattern classification approach based on whole brain-wide rsFC changes (namely more features) (11), although there is no direct statistical comparison. These findings together suggest that brain regions implicated in processing of social and affective domains maybe of greater clinical relevance. Note that although alterations in homotopic inter-hemispheric rsFC have also been found in other disorders such as schizophrenia (84), major depression (85), substance dependence (86), and Parkinson's disease (87), a consistent pattern of decreased homotopic interhemispheric rsFC has only been found in ASD in most of the previous studies across children, adolescents and adults. In other developmental disorders, increased rather than decreased homotopic rsFC has been observed in children with attention-deficit hyperactivity disorder (93) and both increased and decreased homotopic rsFC have been reported in children with Tourette's syndrome (94). Thus, although decreased inter-hemispheric rsFC may not be a biomarker specific for ASD, it may still be a promising biomarker for ASD diagnosis although probably in conjunction with other measures.

## Corpus Callosum and Inter-hemispheric rsFC in ASD

**Figure 2:**
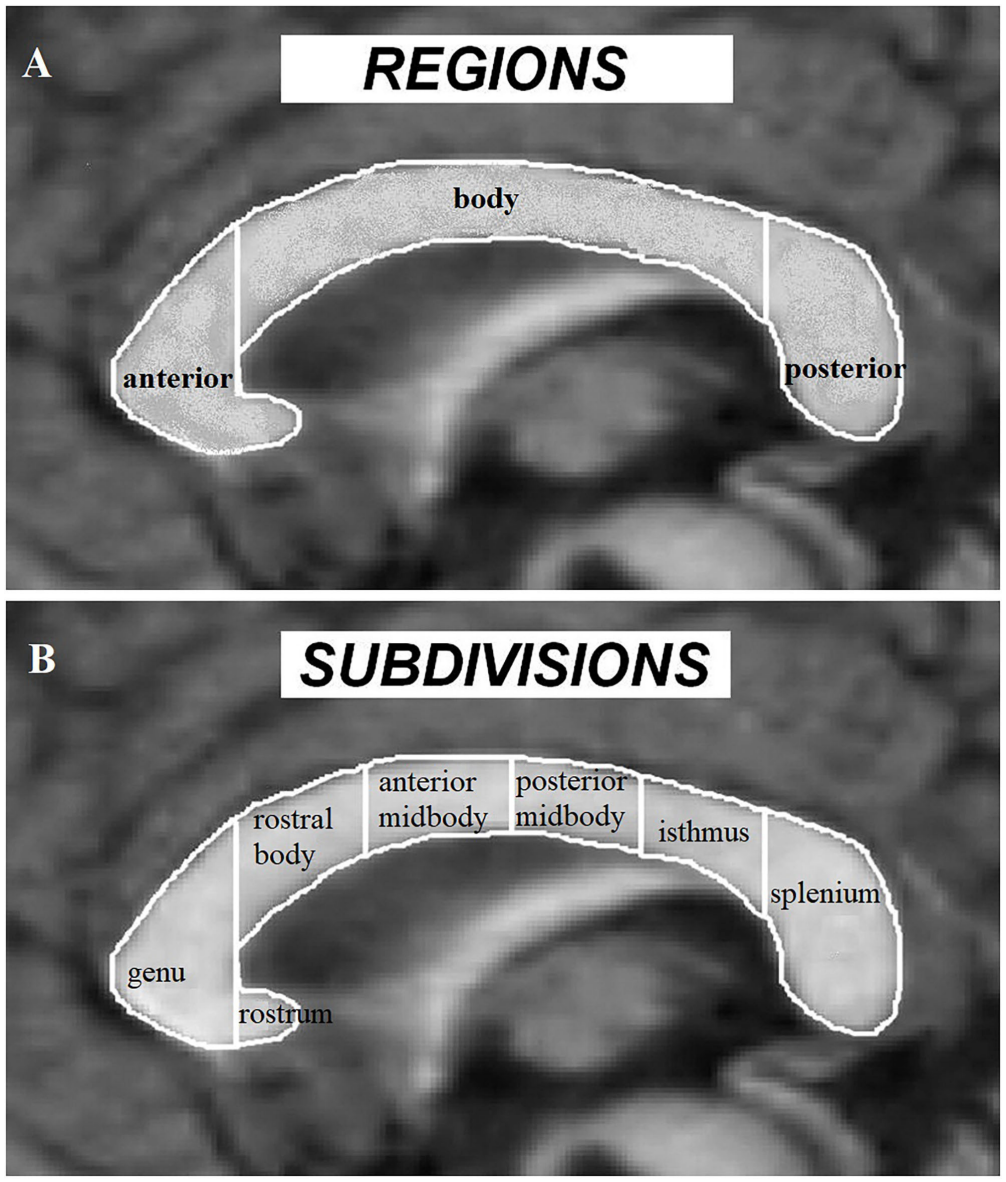
Corpus callosum regions **(A)** and Witelson subdivisions **(B)** adapted from Frazier and Hardan (37).

As the major white-matter tract between the 2 brain hemispheres, CC serves as a bridge for interhemispheric functional communications. In ASD, CC volume has been proposed as an important index of interhemispheric functional connectivity (37, 46). Early findings on CC volume mainly utilize the traditional morphometric method based on the Witelson partition [Figure 2; (95)]. While these studies have consistently demonstrated an overall reduction of CC volume in ASD subjects with and without intellectual disability (35, 42, 43, 96), evidence on the main contributing subregions for such a global reduction effect has tended to be inconsistent with some studies showing significant decreased volume in the anterior subregions (40) and others reporting decreases in posterior part (35, 43) or the CC body (42), although subjects examined in these studies vary across young children, adolescents and adults (from 3 to 42 years old) and across high and low functioning ASD. Vidal et al. (46) further confirmed a global reduction in CC volume and the anterior part of the CC using traditional morphometric methods and found regional reduction in both the anterior (genu) and posterior (splenium) part of the CC using computational mapping methods, reflecting an impact of analysis methods on revealing which specific CC subregions exhibit alterations in ASD. Based on a voxel-based morphometric (VBM) whole brain analysis, a volume reduction has only been found in the posterior CC in ASD [splenium and isthmus; Waiter et al. (47)]. Another VBM study that specifically focused on the CC, has also revealed reduced volume in the posterior (splenium) and additionally in the anterior part (genu and rostrum) of the CC (34). Thus, the regionally restricted (ROI) approach in the latter study may be of greater sensitivity compared to analyses corrected at the whole brain used in the former one. A volume reduction in the anterior and posterior regions of the CC in ASD has also been confirmed by a VBM analysis in a more recent study using the ABIDE database, in which gender, age, IQ, and total brain volume were controlled as potential confounding variables (23). However, using a surface-based approach, Haar et al. (39) did not observe any significant changes in global CC volume in ASD using the ABIDE database, whereas they did find decreased volume in the central part of CC with a small effect size after segmenting the CC into 5 equal parts. Morphometric CC changes in the middle (midbody) and posterior (isthmus and splenium) part have also been confirmed by a diffusional kurtosis imaging study examining axonal density and caliber (45). Finally, another study using the ABIDE database, found no significant CC volume changes based on a more complex model-based analysis method but that CC scaled non-linearly with brain volume, as reflected by large brains having a proportionally smaller CC (41). Meta-analysis studies can provide us with more comprehensive and robust insight on ASD-associated CC changes. In one meta-analytic study ASD individuals were reported to exhibit a general CC volume reduction with the reduction magnitude decreasing from the anterior to posterior part (37). A subsequent meta-analysis has also suggested a group difference in CC volume between the ASD and the TD control groups, but this effect was highly underpowered due to the small sample sizes used in the included studies (41).

In addition, diffusion-weighted imaging (DWI) studies have also revealed CC abnormalities in ASD, as reflected by a general pattern of reduced fractional anisotropy (FA) and concomitantly increased mean diffusivity (MD) or radial diffusivity (RD) in ASD compared to TD controls. This pattern has been found not only in the total CC or across all subregions including the anterior (genu), body, and posterior (splenium) part of CC (49, 52, 58, 64, 65, 67–69), but also in specific CC subregions such as in the genu and body (49, 53, 62), the genu/rostral body (56, 63), the posterior midbody and isthmus (60), and the splenium of CC (50, 57) in high/low-functioning ASD individuals across different age ranges, including children, adolescents, and adults. This has also been confirmed by a diffusion tensor imaging meta-analysis study (51). While having confirmed this pattern of FA changes in ASD children, Barnea-Goraly et al. (54) further revealed that this pattern was shared between ASD children and their unaffected siblings, indicating that FA changes may represent a neural marker reflecting an increased genetic risk or generally increased vulnerability for ASD. Of note very young children with ASD exhibit a different pattern of alterations, such that increased FA in genu and splenium of CC have been found in ASD children aging from 1.8 to 3.3 (55) and an increase in FA but a decrease in RD in the genu and midbody of CC have been found in a slightly older sample (mean age 3.2 ± 1.1) with no changes in MD (70). Moreover, another study did not detect FA changes in adults with ASD (66). Thus, apart from these latter findings the majority of studies converge on a decrease in FA but an increase in MD and RD of CC in ASD. A longitudinal study has further revealed an atypical developmental trajectory of FA of the genu and body of CC before the age of 10 in ASD (67). In contrast, examination of axial diffusivity (AD) revealed somewhat inconsistent results in ASD, with some studies demonstrating significantly higher AD (52) or lower AD either across all 3 subregions (68) or specifically in the body or genu of CC (54, 64) in ASD and others reporting no significant AD differences (49, 70). With respect to associations between diffusion tensor imaging alterations and ASD symptom severity, FA in CC has been found to be negatively correlated with social impairment in ASD as measured by the Social Responsiveness Scale completed by Parents (SRS-P), while alterations in MD and RD have been found to be positively correlated with SRS-P scores (52). In contrast, no significant correlations between FA and diffusivity indices and symptom severity as measured by ADOS have been observed (49, 54, 68). In addition to lower FA and higher diffusivity (as measured by apparent diffusion coefficient), longer fiber length, higher fiber density but a lower fiber number of CC have been reported in ASD children (59, 61), with the fiber number in the anterior CC being negatively correlated with scores on the Childhood Autism Rating Scale (59).

Given that both interhemispheric rsFC and CC volumes exhibit a decreased pattern in ASD individuals, there are also a few studies which have investigated possible relationships between these 2 measures. An absence of significant correlations between the homotopic interhemispheric rsFC and CC volumes has been reported both in a study based on a moderate sample size (53 ASD vs. 39 TDs) (22) and in another larger one using the ABIDE database (23). However, no studies to date have explored the associations between overall interhemispheric rsFC (i.e., not only the homotopic ones) and either CC volumes or DWI indices. Pattern classification analyses based on anatomical abnormalities including variations in CC volumes have reported relatively low classification accuracies of <60% using the ABIDE database (39), which is much lower than pattern classification accuracy based on interhemispheric rsFC changes (23), indicating that anatomical abnormalities measured by conventional MRI protocols are probably too coarse to be a sensitive and robust diagnostic biomarker.

Taken together, although there are some studies which have found no significant CC volume or DWI indices changes in ASD patients (36, 38, 44, 66, 70), the majority of studies have provided converging evidence for decreased CC volumes and FA but increased MD or RD in ASD, particularly those studies based on the traditional morphometric, VBM and DWI methods, although findings on the involvement of specific CC subregions have been less consistent. The heterogeneity of findings on CC volume and DWI indices could be due to differences in sample size, age range, gender, and subject phenotypes [e.g., with and without intellectual disability/high vs. low functioning; cf., (36, 38, 44, 51, 57)]. Differences in analytical approaches and whether potential confounding covariates (e.g., age, gender, IQ, and total brain volume) are well-controlled may also have contributed to these inconsistencies [cf., (34, 40, 43)]. Furthermore, in a longitudinal study of infants (6–24 months), in which subjects were much younger than in all of the studies reviewed above, increased CC area and thickness, particularly robust in the anterior CC, have been found for ASD infants at 6 and 12 months and associated with repetitive behaviors shown at the age of 24 months (48). Similarly, different patterns of FA and RD of CC have been found in very young children with ASD as reviewed above (55, 70). Thus, the developmental trajectory of CC, similar to the aberrant developmental trajectory of the homotopic rsFC (88), should be taken into consideration when interpreting these findings.

## Conclusion and Future Directions

In summary, we have reviewed the literature on inter-hemispheric rsFC and structural CC changes in ASD. While evidence on these differences are heterogeneous, due undoubtedly to some extent to variations in sample size, age range, gender, subject phenotypes and even whether potential confounding covariates are well-controlled, there are nevertheless converging findings on reduced interhemispheric (including homotopic) rsFC in large-scale brain networks particularly in the posterior hubs in the DMN, on reduced volumes in the anterior and posterior CC, and on decreased FA and increased MD or RD across CC subregions. Correlations between the inter-hemispheric rsFC changes and symptom severity in social impairments and the superior classification performance in distinguishing ASD subjects from TD controls based on the interhemispheric rsFC changes together suggest that inter-hemispheric rsFC changes, a typical form of long-distance connection, may be a more promising biomarker for ASD diagnosis than whole brain wide rsFC and structural abnormalities. Future studies will also need to focus on investigating whether these biomarkers can be applied to other novel samples with a high generalizability if we are to progress toward the ultimate goal of such biomarkers having utility at an individual as opposed to a group level. Additionally, the specificity of interhemispheric rsFC changes in ASD compared with other childhood disorders needs to be more fully established.

More future studies are also required using different multimodal brain imaging and behavioral approaches to better establish the functional consequences of dysfunctional inter-hemispheric connectivity in ASD. Furthermore, given evidence in both humans and animal models for considerably potential for functional recovery in interhemispheric rsFC following CC damage or impaired development it will be important to establish if this could be exploited therapeutically. In addition, based on previous finding that the development or microstructure of CC can be modulated by factors such as sex hormones (97, 98), age (99), prenatal inflammation (100) and mutation of specific genes (101, 102), future studies should aim at disentangling the potential contributions of these factors to CC alterations in ASD.

There has also been considerable interest in identifying sub-types in ASD, as in other disorders, and moving forward it will clearly be important to do this either based on brain- or behavioral-based subtyping. Previous research on brain-based biomarkers has also been focused almost exclusively on high functioning ASD and on adolescents and adults and therefore there is an urgent need for more studies and hopefully databases which include low-functioning individuals and particularly young children where early diagnosis and therapeutic interventions are potentially of greatest importance. Finally, to make findings across studies and databases more comparable and reproducible, it is also important for future studies follow standardized data processing procedures with potential confounding covariates being well-controlled [cf., (103, 104)].

## Author Contributions

All authors contributed to the information and ideas presented in the review and writing of the manuscript.

## Conflict of Interest

The authors declare that the research was conducted in the absence of any commercial or financial relationships that could be construed as a potential conflict of interest.
